# Huge Temperature-Induced Increase in Chemical Resistance of Solution-Processed Amorphous Thin Films along the As_3_S_7_–MoS_3_ Tie-Line and Its Structural Explanation

**DOI:** 10.1021/acsomega.5c05113

**Published:** 2025-10-03

**Authors:** Jiri Jancalek, Roman Svoboda, Bozena Frumarova, Milos Krbal

**Affiliations:** 495954 University Pardubice Faculty of Chemical Technology, Center of Materials and Nanotechnology, Nám. ČS Legií 565, Pardubice 532 10, Czech Republic

## Abstract

Amorphous chalcogenide thin films have been widely studied for use in lithography as a photoresist due to significant changes in etching rates caused by photoinduced structural changes upon exposure to light. Here, we report on the role of the transition metal molybdenum contained in MoS_3_ on the significantly enhanced thermal stability and chemical resistance of As_3_S_7_–MoS_3_ thin films deposited by a sol–gel method. We demonstrate that the dependences of etching rates of As_3_S_7_–MoS_3_ thin films in a solution consisting of 1 vol % *n*-butylamine in dimethyl sulfoxide on the annealing temperature exhibit two linear regimes with a sudden change of their slopes at 160 °C. The first regime, below 160 °C, is characterized by a significantly lower chemical resistance of As_3_S_7_–MoS_3_ thin films in comparison with As_3_S_7_ thin films, while the second regime manifests a gradual decrease in etching rates by 4 orders of magnitude in a range of annealing temperatures from 160 to 200 °C. We explain both trends using optical properties, differential scanning calorimetry, and attenuated total reflection results. We propose that the vast, 4-orders-of-magnitude difference in the etching rate between as-deposited As_3_S_7_–MoS_3_ and annealed thin films at 200 °C can be successfully applied as a resist layer in wet lithography.

## Graphical abstract

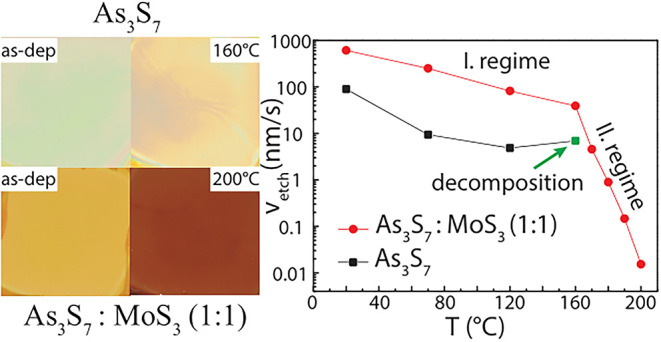

## 1 Introduction

Amorphous chalcogenide solids possess unique properties due to the presence of so-called lone-pair electrons at the top of the valence band, which are sensitive to temperature,^1^ photons,^2,3^ electric fields,^4,5^ etc., causing either reversible or irreversible changes in the local bonding. The local structural modifications in the amorphous network induce significant changes in the physicochemical properties, which can be easily controlled and employed in many applications. For instance, they have been commercially implemented in phase change memories,^4^ X-ray medical image sensors,^6,7^ holograms,^8^ and grayscale lithography.^9^ In addition, the significant contrast in optical and electrical properties between the two states has been recently also adopted in neuro-inspired computational devices,^10,11^ sensors,^12^ nanophotonics,^13,14^ etc. Chalcogenide glasses are, in most cases, a mixture of chalcogen atoms with other elements that have p-bonding orbitals. The development of new materials with tunable parameters can be achieved by, for example, the incorporation of a transition metal into an amorphous chalcogenide network. The advantage of this can be found in either unpaired d-electrons, increasing the magnetic properties,^15^ or empty d-orbitals, which can form bonds with lone-pair electrons of a chalcogenide atom, the so-called donor–acceptor (dative) bonds. In the latter case, the amount of lone-pair electrons in amorphous chalcogenides can be easily optimized for designed applications.^16^ On the other hand, the high melting point of most transition metals or their chalcogenide compounds makes it impossible to synthesize transition metal-doped chalcogenide bulk glasses by the melt-quenching method. Moreover, the apparent segregation of transition metal chalcogenides, occurring already in the amorphous phase, emphasizes the above-mentioned drawback and prevents their uniform distribution over the entire glass volume.^17,18^ Consequently, amorphous transition metal-based chalcogenide solids can only be utilized as thin films prepared by physical vapor deposition (PVD) techniques^19–21^ or a sol–gel method.^22^ In lithography, it is essential that there is a significant difference in the solubility of the exposed and unexposed portions of the resist layer in the photoresist developer. Since the chalcogenide thin films are extremely resistant to acid etching solutions, the applied developers are either inorganic alkaline solutions^19,23^ or organic amine-based solutions.^9,19,24,25^ Although the use of thin amorphous chalcogenide thin films in wet etch lithography has been widely studied for several decades,^25,26^ the application of transition metal-based chalcogenides has been limited to Ag-doped amorphous thin films,^9,19,23,27,28^ reporting that the etching rate of Ag-doped layers could be reduced by several orders of magnitude or, depending on the etchant chosen, even completely blocked by the presence of silver in a thin layer. The role of chalcogen on the etching resolution was clearly described on Ag-doped As–S–Se thin films.^19^ Using a water solution of NaCN as a developer, the Ag–As_33_S_67_ thin film is exclusively a positive resist compared to the solely negative resist of Ag–As_33_Se_67_. The Ag–As_33_S_67–y_Se_*y*_ ternary system can be used as a positive or negative resist, with a negative etching process being preferred with increasing Se content, which is further accentuated by the presence of Ag. Recently, a new class of transition metal-based chalcogenide thin films has been developed along the As_2_S_3_–MoS_3_ tie-line.^22^ The wide variability between As_2_S_3_ and MoS_3_ contents in amorphous thin films can help control the chemical resistance of these films to organic amine-based solutions. In this paper, we demonstrate the role of the transition metal molybdenum contained in MoS_3_ on the significantly enhanced thermal stability and chemical resistance of As_3_S_7_–MoS_3_ thin films deposited by a sol–gel method. We show the dependences of etching rates on the annealing temperature of As_3_S_7_–MoS_3_ thin films, occurring in two linear regimes with significantly different slopes, varying at 160 °C. The abrupt change between etching modes is clarified by using the results obtained by the attenuated total reflection (ATR) and differential scanning calorimetry (DSC) techniques.

## 2 Experimental Section

### 2.1 Bulk Glass Synthesis, Solution Preparation, and Thin-Film Deposition

A bulk As_3_S_7_ glass was synthesized from As (5N, HiChem spol.) and S (5N, HiChem spol.) by the standard melt-quenching technique.^22^ Prior to deposition of thin films, two 0.02 M stock solutions were prepared by dissolving 0.9 g of As_3_S_7_ and 1.3 g of (NH_4_)_2_MoS_4_ (99.97%, Sigma-Aldrich) in 10 mL of *n*-propylamine (PA, ≥99%, Sigma-Aldrich).^22^ The designed compositions were then engineered by mixing the two stock solutions in ratios of 5:1, 2:1, and 1:1, which were then thoroughly shaken and left to stand for 24 h to react with each other. Thin films were deposited onto square ≈ 2.5 × 2.5 cm^2^ soda-lime microscope glass substrates by stationary spin-coating. 100 μL of the solution was pipetted onto the substrate and subsequently centrifuged at 3500 rpm for 30 s. As-prepared thin films were dried at room temperature in an argon vacuum atmosphere (three cycles of 5N argon purging and then evacuation at residual pressure ≈ 1 Pa) for 1 h in a vacuum furnace. After drying, the films were annealed at 70, 120, 160, 170, 180, 190, and 200 °C for 2 h under an Ar environment at a residual pressure of 1 Pa. Thin films were then stored in a nitrogen-filled glovebox to prevent possible hydrolysis due to atmospheric humidity.

### 2.2 Thin-Film Characterization

The optical transmittance spectra of the prepared thin films were recorded by using a Shimadzu two-beam (ultraviolet–visible) UV–vis–NIR spectrophotometer in the wavelength range from 190 to 2000 nm with a measurement step of 1 nm. The thickness and refractive index were evaluated from optical transmittance spectra using a combination of the Swanepoel model^29^ with the Wemple–DiDomenico parametrization.^30^ The amorphous character of thin films was confirmed by X-ray diffraction using a diffractometer (Empyrean Malvern Panalytical) operating in the 2 theta/omega mode to fully satisfy the Bragg–Brentano geometry. The wet etching kinetics of thin films were investigated by an interference method using a fiber spectrometer EPP2000 (StellarNet) with a tungsten halogen lamp SL1 (StellarNet) as a source of the probing light. The etching solution was a mixture of 1 vol % butylamine (BA, 99.5%, Sigma-Aldrich) in dimethyl sulfoxide (DMSO, p.a., Lach/ner). The risk of hydrolysis of the etching bath or samples was reduced by slowly blowing argon above the surface of the etching bath. The conditions for ammonium salt removal were tested by means of DSC. In this regard, the heat-flow differential scanning calorimeter Q2000 DSC (TA Instruments) was used, equipped with a cooling accessory, an autolid, an autosampler, and T-zero technology. The As_3_S_7_ and As_3_S_7_–MoS_3_ samples in the powder form (thin films scraped off of the standard microscopy slides) were inserted in low-mass aluminum DSC pans and hermetically sealed. The DSC measurements were performed as simple heating scans at 20 °C.min^–1^. Attenuated total reflection (ATR) spectra were measured by using a Vertex 70v Fourier transform infrared spectrometer (Bruker) with a diamond crystal ATR adapter.

## 3 Results and Discussion

Prior to studying the wet etching characteristics of thin films along the As_3_S_7_–MoS_3_ tie-line, including an explanation of all effects, we performed necessary analyses describing the prepared thin films. Photographs of thin films of As_3_S_7_ and As_3_S_7_–MoS_3_ (in a 1:1 molar ratio) deposited by spin-coating and annealed at 70, 120, 160, 170, 180, 190, and 200 °C are demonstrated in Figure 1. At the first glance, the As_3_S_7_ thin films were found to be stable up to an annealing temperature of 120 °C, while thin-film decomposition was observed at 160 °C. Annealing of As_3_S_7_ thin films at temperatures exceeding 160 °C resulted in the complete decomposition and evaporation of the layer itself. In contrast, the presence of MoS_3_ in As_3_S_7_ significantly increases the thermal stability of prepared thin films until 200 °C (the highest studied temperature), with no observed redeposit of the material after annealing. Visually, the color of the thin films darkens with increasing annealing temperature, which is attributed to the gradual removal of excess solvent and decomposition of ammonium salts. The result is a gradual increase in the density of the prepared thin layers.^31^ The significance of this effect is discussed later.

**Figure 1:**
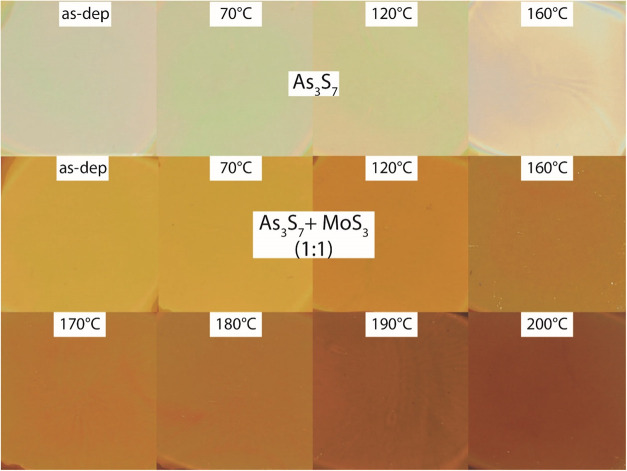
As_3_S_7_ and As_3_S_7_–MoS_3_ thin films prepared by spin-coating and annealing in the range of temperatures from room temperature (dry) to 200 °C.

The amorphous nature of all thin films was confirmed by X-ray diffraction analysis, as shown in Figure 2. This result is in good agreement with the fact that As–S thin films can be easily prepared amorphous,^22,32^ and MoS_3_ exists only in the amorphous state, which is well stable up to 200 °C.^33^

**Figure 2:**
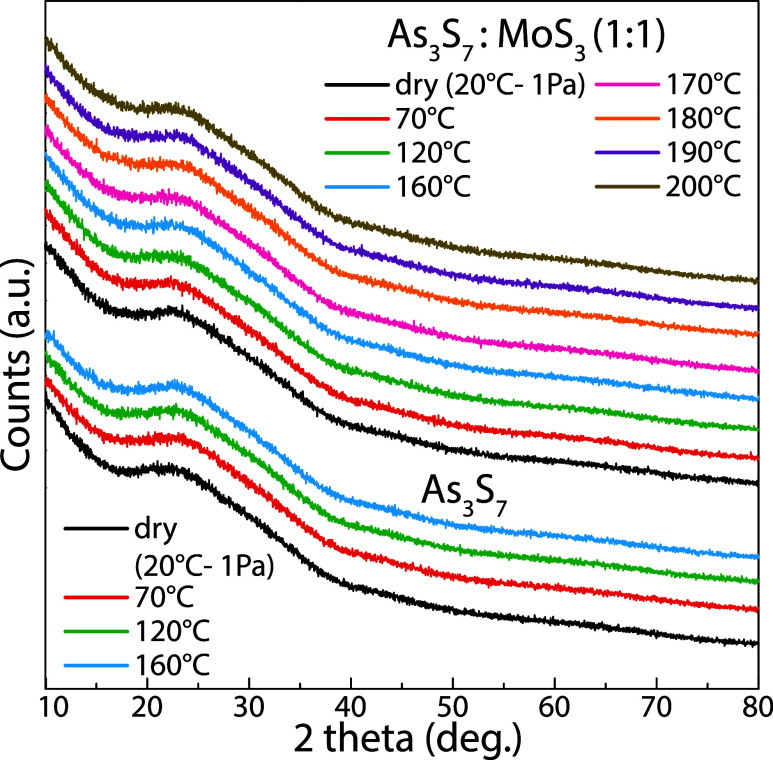
XRD patterns of spin-coated As_3_S_7_ and As_3_S_7_–MoS_3_ thin films in the range of temperatures from room temperature (dry) to 200 °C.

Optically homogeneous transmittance spectra of thin films are a vital prerequisite for wet etching experiments to measure the change in thin-film thickness by an interferometric method. The optical transmittance spectra of thin films are shown in Figure 3 A and B, demonstrating that the prepared layers are well transparent and, at the same time, do not significantly scatter light over the entire measured spectral range. It is also apparent from Figure 3 A and B that the short-wavelength absorption edge is gradually red-shifted with increasing annealing temperature, indicating a decrease in the value of the optical band gap (E_g_^opt^). Similarly, the fringe minima deepen and, at the same time, shift to lower wavelengths, corresponding to an increase in the refractive index and a decrease in the layer thickness, as illustrated in Figure 3 C and D. All of these facts manifest the gradual release of the residual solvent from the thin layers together with the decomposition of ammonium salts.^22^ Finally, the thermal instability of As_3_S_7_ thin films annealed at 160 °C is best illustrated in Figure 3 D, showing a rapid decrease in the relative thickness of the annealed As_3_S_7_ thin film in the temperature range from 120 to 160 °C compared to the relative thickness of the As_3_S_7_–MoS_3_ thin film studied over the entire temperature range, which decreases linearly.

**Figure 3:**
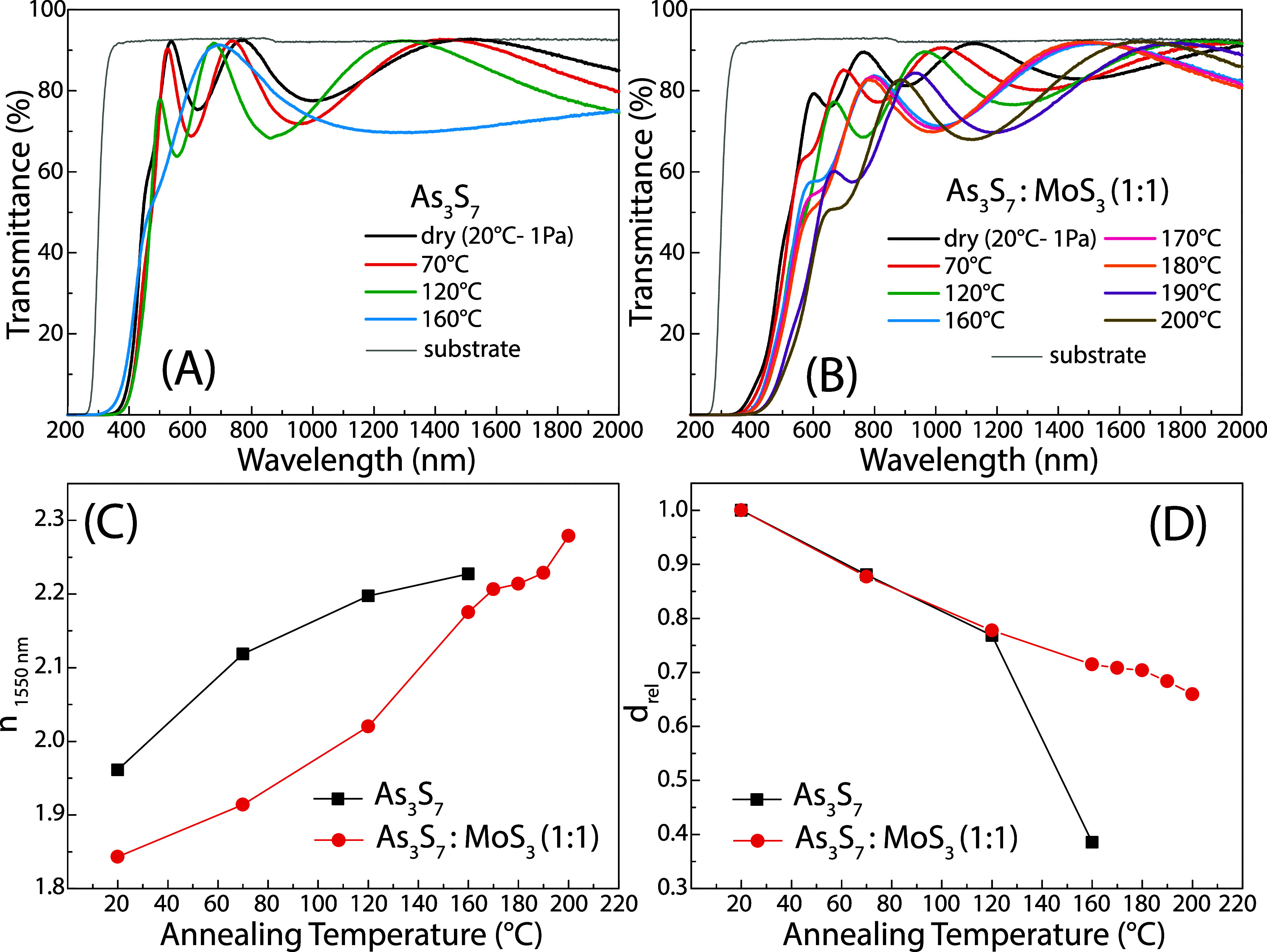
Optical transmittance of spin-coated (A) As_3_S_7_ and (B) As_3_S_7_–MoS_3_ thin films annealed in the range of temperatures from room temperature (dry) to 200 °C. (C) Calculated refractive index at 1550 nm (n_1550 nm_) and (D) relative thickness d_rel_ of As_3_S_7_ and As_3_S_7_–MoS_3_ thin films for different annealing temperatures.

The chemical resistance of As_3_S_7_ and As_3_S_7_–MoS_3_ thin films, which were dried under vacuum at room temperature and gradually annealed at 70, 120, 160, 170, 180, 190, and 200 °C, was investigated in a solution consisting of 1 vol % BA in DMSO. The obtained dependences of the etching rate on the annealing temperature of thin films are provided in Figure 4. Selected examples of monitored etching kinetics as a function of the relative layer thickness and transmission of the first interference maximum over the etching time are shown in Figure S1 of the Supporting Information.

**Figure 4:**
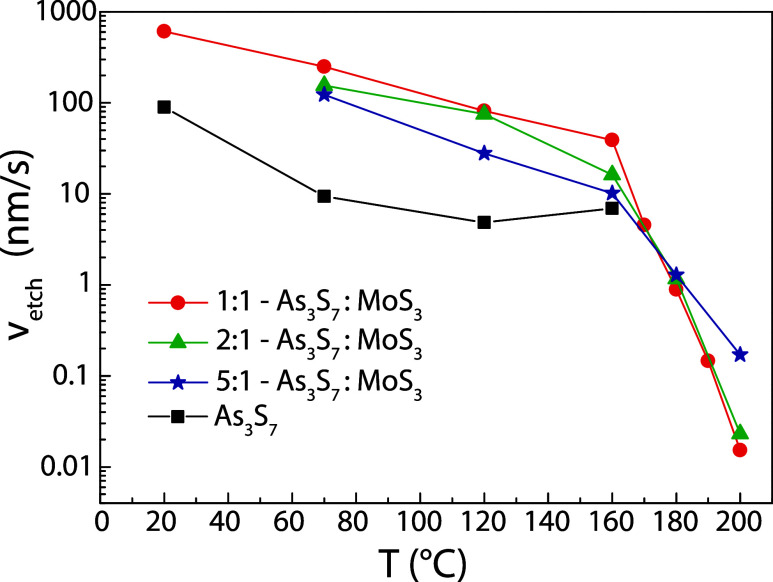
Etching rates of spin-coated As_3_S_7_ and As_3_S_7_–MoS_3_ thin films annealed in the range of temperatures from room temperature (dry) to 200 °C. The error of determination due to variations in the thickness of thin films is approximately 5%.

We show that the dependences of etching rates on the annealing temperature of As_3_S_7_–MoS_3_ thin films proceed in two linear regimes, which are suddenly changed at 160 °C. In the first regime, below 160 °C, the chemical resistance of As_3_S_7_–MoS_3_ thin films is about 1 order of magnitude lower than As_3_S_7_ thin films. The second regime is characterized by the rapid decrease in etching rates by up to 4 orders of magnitude in the range of annealing temperatures from 160 to 200 °C. Furthermore, it can be seen that the concentration of MoS_3_ in As_3_S_7_ affects the etching kinetics of the layers in both regimes, wherein in the first step, the etching rate increases with increasing MoS_3_ concentration, while it slows noticeably in the second step. Interestingly, the etching rate dependences of thin As_3_S_7_ films with different MoS_3_ concentrations cross at the same annealing temperature of 175 °C, which is called the isosbestic point, indicating that as the concentration of MoS_3_ increases, the solubility of the layer decreases in direct proportion at the same temperature.

To better understand the differences in etching characteristics of prepared thin films, we performed detailed analyses of the thin-film structure and annealing-induced structural changes studied by DSC and ATR techniques, respectively. As shown in Figure 5, the DSC curves are shown for the As_3_S_7_ and As_3_S_7_–MoS_3_ samples annealed at different temperatures for 2 h. The DSC curve for the pure As_3_S_7_ films shows a very weak glass transition effect around 145 °C, which increases with annealing to 170 °C. This shift indicates a significant change in the structural ordering (toward a more compact layer) of the glassy structure, which is most probably due to the removal of the residual solvent and the subsequent cancellation of the *T*_g_-lowering plasticization effect. At higher temperatures (above 200 °C), the As_3_S_7_ samples exhibit exothermic degradation of the glassy structure.

**Figure 5:**
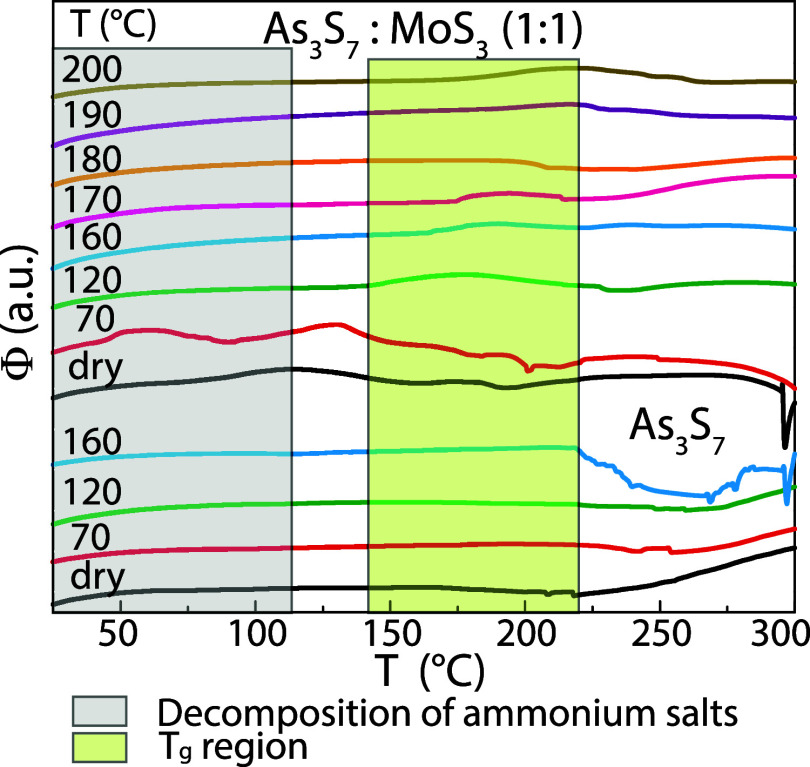
DSC curves of spin-coated As_3_S_7_ and As_3_S_7_–MoS_3_ thin films annealed in the range of temperatures from room temperature (dry) to 200 °C measured with a heating rate of 20 K/min.

The as-prepared As_3_S_7_–MoS_3_ samples show a complex thermal behavior during heating, as evidenced in Figure 5, exhibiting several endothermic and exothermic effects, which correspond to the decomposition of the ammonium salts and the release of the products of these reactions. This behavior persists even at low-temperature (70 °C) annealing since the degradation kinetics proceeds in multiple steps with high activation energy (the processes are highly temperature-dependent). Most decomposition products appear to be removed during the annealing at 120 °C, where the corresponding (concurring) DSC heating results in a single exothermic effect with the onset at 140 °C. Annealing at higher temperatures (160 °C) leads to a shift of the onset to 165–170 °C; further increases of the annealing temperature (tested up to 200 °C) then do not further increase the onset of this single exothermic effect. Based on the possible physicochemical reactions and transformations in the annealed As_3_S_7_–MoS_3_ samples, the following findings should be taken into account while considering the nature of this exothermic effect: (1) crystallization of the amorphous phase can be ruled out based on the X-ray diffraction (XRD) data (Figure 2) and (2) further decomposition of the ammonium salts can also be ruled out, as the exothermic effect does not considerably decrease in magnitude with the annealing temperature, and the thermal-kinetic complexity of the DSC record (attributed to the salt decomposition) entirely vanished at 120–160 °C. Note that the ATR results (reported below) have confirmed that no ammonium salts are present after annealing at a temperature of 160 °C. This leaves the above-discussed exothermic effect to be associated with the interaction between the As_3_S_7_ and MoS_3_ structural units, resulting in the formation of the bridging (S–S)^2–^ units that significantly strengthen the amorphous network, causing the decrease of the (wet) etching rates. Note the temperature correspondence of the onset of this interaction (165 and 170 °C, as shown in Figure 5) with the initiation of the sharp decrease of etching rates shown in Figure 4. It is also worth noting that this interaction is slow with a catalytically limited transformation rate (exhibiting a similar onset and magnitude of the DSC peak even after 2 h annealing at 200 °C).

Structural changes in thin films during annealing are shown in Figure 6. The ATR spectrum of as-deposited As_3_S_7_ for the wavenumber region from 700 to 50 cm^–1^ forms vibration bands at 290, 310, 343, 366, and 412 cm^–1^ and above 500 cm^–1^. Vibration bands at 290 and 310 cm^–1^ are assigned to AsS_3_ pyramidal units,^34,35^ 343 cm^–1^ to a joint contribution of AsS_3_ pyramidal units and As_4_S_4_,^36^ and 366 cm^–1^ to As_4_S_4_.^34,36^ Vibration bands at 412 cm^–1^ and above 500 cm^–1^ can be attributed to a residual n-propylamine solvent and ammonium salts. The ATR spectrum of as-deposited As_3_S_7_–MoS_3_ in the ratio 1:1 contains additional vibration bands at 437, 458, and 520 cm^–1^. In more detail, the vibration band at 437 cm^–1^ is attributable to the asymmetric stretching vibration of the Mo = S units in the MoS_2_^–4^ anions,^37^ 458 cm^–1^ is assigned to the apical sulfur atom in the S–Mo_3_ triangular unit,^37^ and 520 cm^–1^ corresponds to the terminal (S–S)^2–^ units.^37^ It is evident from Figure 6 that a significant portion of the residual solvent is removed along with the decomposition of a principle amount of ammonium salts between 70 and 160 °C, which is in good agreement with a previous study.^31^ Above the latter temperature, a new vibration band at 500 cm^–1^ emerges in the ATR spectrum and gradually increases its intensity with increasing temperature until 200 °C. This vibration mode is attributable to the bridging (S–S)^2–^ units,^37^ which further strengthen the amorphous network. Strikingly, the observed change in the inner structure of the annealed films at 160 °C is in excellent agreement with the abrupt switch between the two etching regimes. We, therefore, suggest that the presence of organic ammonia-based compounds (the residual solvent and ammonium salts including the Mo = S units in the MoS_2_^4–^ anions) in the amorphous thin film increases the dissolution of As_3_S_7_–MoS_3_ compared to pure As_3_S_7_ thin films in the amine-based etching solvent. On the other hand, forming the bridging (S–S)^2–^ units significantly improves the etching resistance of As_3_S_7_–MoS_3_ thin films. In addition, we assume that the bridging (S–S)^2–^ units can also contribute to the increased thermal stability of the As_3_S_7_–MoS_3_ thin films.

**Figure 6:**
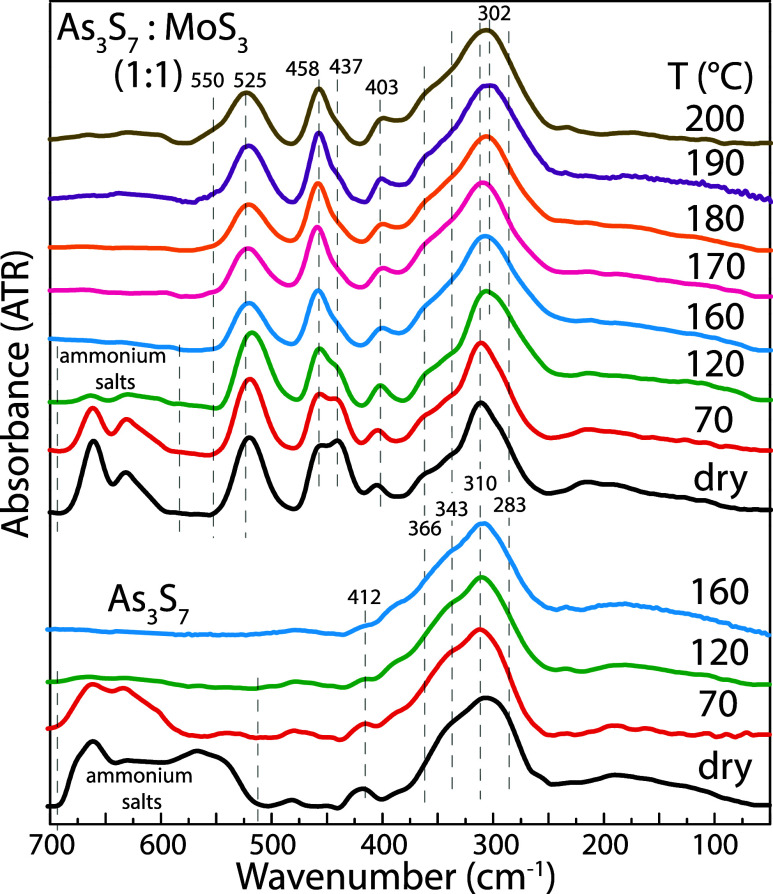
ATR infrared spectra of spin-coated As_3_S_7_ and As_3_S_7_–MoS_3_ thin films annealed in the range of temperatures from room temperature (dry) to 200 °C.

Our results illustrate the applicability of Mo-containing amorphous chalcogenide thin films in wet lithography as a negative resist due to the giant difference, almost 5 orders of magnitude, in the etching rate between the as-deposited As_3_S_7_–MoS_3_ thin films and the counterparts annealed at 200 °C. We predict that locally modified structures in As_3_S_7_–MoS_3_ thin films, e.g., by Joule heating or laser exposure, are protected from etching in amine-based solutions.

## 4 Conclusions

In this article, we reported the role of MoS_3_ on the significantly enhanced thermal stability and chemical resistance of As_3_S_7_–MoS_3_ thin films deposited by a sol–gel method. We demonstrated that As_3_S_7_ thin films decompose at 160 °C, whereas As_3_S_7_–MoS_3_ thin films are thermally stable up to 200 °C. We showed that the dependences of etching rates on the annealing temperature of As_3_S_7_–MoS_3_ thin films proceed in two linear regimes, which are abruptly changed at 160 °C. The first regime, below 160 °C, is characterized by a significantly lower chemical resistance of As_3_S_7_–MoS_3_ thin films in comparison with As_3_S_7_ thin films, and the second regime manifests the gradual decrease in etching rates by as much as 4 orders of magnitude in the range of annealing temperatures from 160 to 200 °C. We suggested, based on the results of DSC and ATR studies, that the presence of organic ammonia-based compounds, including the Mo = S units in the MoS_2_^–4^ anion in the amorphous thin film existing below 160 °C, accelerates the dissolution of As_3_S_7_–MoS_3_ compared to pure As_3_S_7_ thin films in an amine-based etching solvent and the formation of the bridging (S–S)^2–^ units evolving above 160 °C on the other hand significantly improves the etching resistance of As_3_S_7_–MoS_3_ thin films under the same etching conditions. We proposed that As_3_S_7_–MoS_3_ thin films can find their potential application as a negative resist in wet lithography, demonstrating the considerable difference, almost 5 orders of magnitude, in the etching rate between as-deposited As_3_S_7_–MoS_3_ and annealed thin films at 200 °C.

## Supplementary Material



## Notes

Time dependences of relative thickness during wet etching for As3S7 (dry and annealed at 70 and 160°C) and As3S7/MoS3 1:1 (dry and annealed at 70, 160, 180 and 200°C) (PDF)

## Abbreviations

PA: n-propylamine
BA: *n*-butylamine
DMSO: dimethyl sulfoxide
PVD: physical vapor deposition
ATR: attenuated total reflection
DSC: differential scanning calorimetry
XRD: X-ray diffraction
