# Supplementary material for: Huge Temperature-Induced Increase in Chemical Resistance of Solution-Processed Amorphous Thin Films along the As3S7–MoS3 Tie-Line and Its Structural Explanation
Source: ACS Omega. 2025 Oct 3;10(40):46928–34. doi: 10.1021/acsomega.5c05113 (PMC12529201; doi:10.1021/acsomega.5c05113)
Supplement: Supplementary file 1 [file ao5c05113_si_001.pdf]

Huge temperature-induced increase in chemical resistance of solution-processed amorphous thin films along the As_3S_7 -MoS_3 tie-line and its structural explanation – supplementary information

*Jiri Jancalek**, *Roman Svoboda, Bozena Frumarova, Milos Krbal*

University Pardubice Faculty of Chemical Technology, Center of Materials and Nanotechnology, Nám. ČS Legií 565, Pardubice, Czech Republic

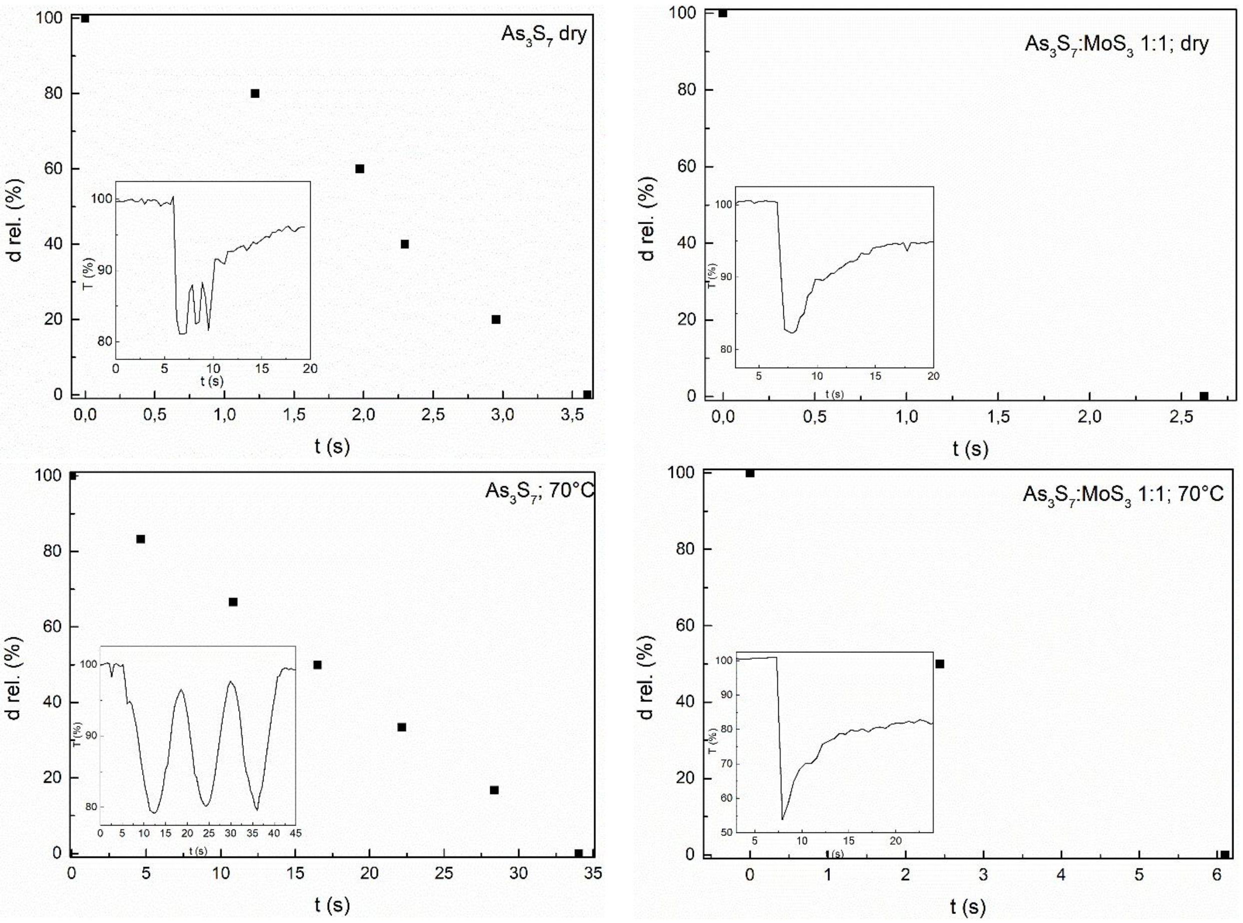

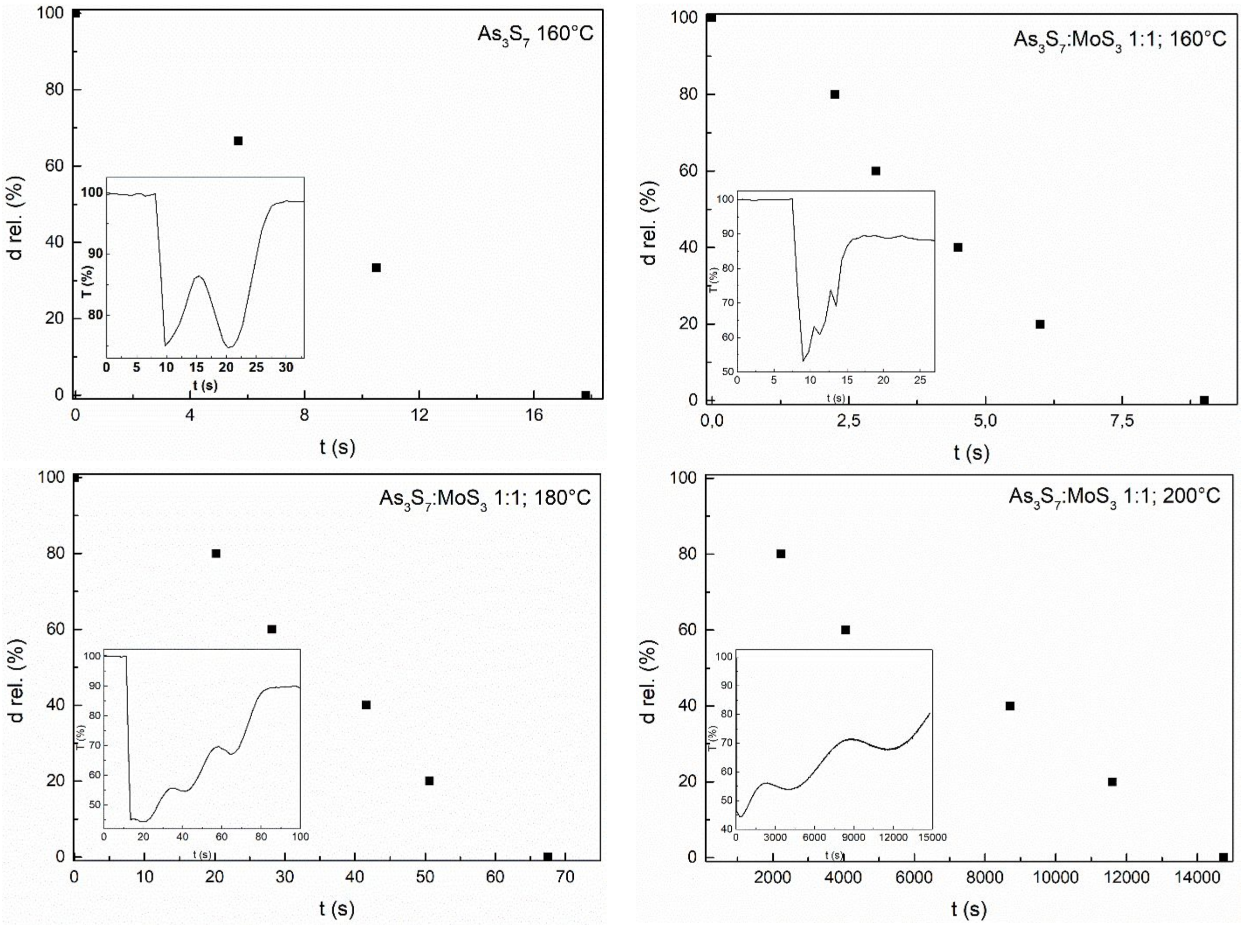

Figure S1: Time dependences of relative thickness during wet etching for As_3S_7 (dry and annealed at 70 and 160°C) and As_3S_7:MoS_3 1:1 (dry and annealed at 70, 160, 180 and 200°C). The inserted graphs represent the time dependence of transmittance at the first interference maxima in the corresponding spectrum.
